# Detection and Quantification of the E6 Oncogene in Bovine Papillomavirus Types 2 and 13 From Urinary Bladder Lesions of Cattle

**DOI:** 10.3389/fvets.2021.673189

**Published:** 2021-05-14

**Authors:** Brígida Kussumoto de Alcântara, Michele Lunardi, Alais Maria Dall Agnol, Alice Fernandes Alfieri, Amauri Alcindo Alfieri

**Affiliations:** ^1^Laboratory of Animal Virology, Department of Veterinary Preventive Medicine, Universidade Estadual de Londrina, Londrina, Brazil; ^2^Multi-User Animal Health Laboratory, Molecular Biology Unit, Department of Veterinary Preventive Medicine, Universidade Estadual de Londrina, Londrina, Brazil; ^3^Laboratory of Veterinary Microbiology, Universidade de Cuiabá, Cuiabá, Brazil; ^4^National Institute of Science and Technology for Dairy Production Chain (INCT-LEITE), Universidade Estadual de Londrina, Londrina, Brazil

**Keywords:** cattle, bovine enzootic hematuria, deltapapillomavirus, BPV2, BPV13, E6 ORF, qPCR

## Abstract

Bovine papillomavirus types 2 and 13 can induce tumors in both the cutaneous and mucosal epithelia of cattle. These viral types are associated with the development of benign cutaneous papillomas and malignant lesions in the urinary bladders of cattle, with the latter being known as bovine enzootic hematuria. Among the viral oncoproteins encoded by *Deltapapillomavirus* DNA, the E6 oncoprotein has an important role in cell proliferation and might be related to cancer initiation and promotion. The aim of this study was to present a standardized SYBR Green-based quantitative PCR for detection and quantification of the bovine papillomavirus 2 and 13 E6 oncogenes in urinary bladder samples from cattle. Twenty-four urinary bladders from cattle displaying tumors (*n* = 12) and normal bladder mucosa (*n* = 12) were tested by quantitative PCR. Of the 12 urinary bladders with tumors, six presented bovine papillomavirus 2 DNA concentrations ranging from 1.05 × 10^4^ to 9.53 × 10^3^ copies/μL, while two had bovine papillomavirus 13 DNA amplified at concentrations of 1.30 × 10^4^ to 1.23 × 10^4^ copies/μL. The healthy bladder mucosa samples were negative for both bovine papillomaviruses. Once the results were confirmed by conventional PCR and direct sequencing, the quantitative PCR assay developed in this study was shown to be a sensitive and specific tool for detecting and quantifying the E6 ORF of bovine papillomavirus 2 and 13 in a variety of clinical samples. Our findings of identification of bovine papillomavirus 2 and 13 DNA in urothelial tumors from cattle suffering from bovine enzootic hematuria agree with data from previous studies, representing the first detection of bovine papillomavirus 13 DNA in malignant bladder lesions of cattle from Brazil.

## Introduction

Bovine papillomaviruses (BPVs) cause benign hyperproliferative lesions of the cutaneous and mucosal epithelia, leading to distressing animal diseases, and considerable economic losses (1). Bovine enzootic hematuria (BEH) is a clinical syndrome characterized by chronic inflammation and the presence of malignant lesions in the urinary bladder of cattle, resulting in intermittent hematuria, anemia, progressive emaciation, and death. Carcinogenesis observed in the urinary bladder of cattle affected by BEH has been associated with synergism between BPV infection and chronic intoxication by bracken fern (*Pteridium aquilinum*) ingestion, which contains mutagenic and carcinogenic principles as quercetin and ptaquiloside. These compounds trigger viral gene expression that results in cell transformation, initiating the progression to malignancy (2, 3). Cases of urinary bladder cancer occur in cattle herds grazing on pasture where bracken fern is spread, with a prevalence as high as 90% in adult animals from some geographic areas endemic for BEH as continental Europe, the Azores Islands, in some regions of Kenya, Brazil, New Zealand, India, and China (3).

BPVs are small oncogenic DNA viruses classified as members of the *Papillomaviridae* family within five different genera: *Deltapapillomavirus* groups BPVs types 1, 2, 13, and 14; BPVs types 3, 4, 6, 9, 10, 11, 12, 15, 17, 20, 23, 24, 26, and 28 in *Xipapillomavirus*; BPVs 5, 8, and 25 belong to the *Epsilonpapillomavirus* genus; BPVs 16, 18, and 22 are classified in *Dyokappapapillomavirus*; and BPV7, which is the only member of the *Dyoxipapillomavirus* genus. BPVs 19, 21, and 27 remain unclassified (4). Classically, BPV2 has been detected in urinary bladder tumors and clinical samples from cattle suffering from BEH in endemic regions for the disease (2, 5–7). Recently, BPV13 was also implicated as a possible causative factor of urothelial tumors in cattle from Italy, in which a high rate of BPV13 DNA detection occurred in urinary bladder tumors, and E5 oncoprotein expression was shown in tumoral tissues (8).

Along with the E5 viral protein, which is believed to be the major oncoprotein of BPVs in the *Deltapapillomavirus* genus, the oncoprotein encoded by the E6 gene was shown to be capable of inducing a full transformation of equine immortal fibroblasts, which are highly involved in the anchorage independence of equine sarcoid fibroblasts and cell invasion (9, 10). The mutagenic potential of the BPV1 E6 recombinant oncoprotein was confirmed by inducing clastogenesis *per se*, which is associated with host genomic instability, suggesting that this viral oncoprotein participates in both cancer initiation and promotion (11).

Most investigations of BPV infection in clinical samples from cattle suffering from chronic BEH were performed based on conventional PCR strategies specific for BPV2 (6, 12–14). However, diverse studies focusing on higher sensitivity and specificity for the detection of BPV DNA based on real-time quantitative PCR analyses presented viral load estimations for BPV2 DNA in samples from bovine bladders with and without tumoral lesions (7, 15, 16). Given the lack of a real-time quantitative PCR technique for the detection and quantification of both BPV2 and BPV13 DNA, the aim of this study was to present a new standardized SYBR Green-based qPCR for the detection and quantification of BPV2 and BPV13 E6 oncogenes in urinary bladder samples from cattle.

## Materials and Methods

### Clinical Samples and DNA Extraction

Twelve urinary bladders with macroscopic lesions were collected from adult bovines from two different farms in a slaughterhouse in southern Brazil. Twelve other urinary bladders without macroscopic lesions were also collected from the same slaughterhouse. Both tumoral and normal bladder samples were divided into several parts. Some parts were fixed in 10% buffered formalin for microscopic evaluation while a fragment was maintained at −80°C until used for molecular analyses. Urinary bladder carcinomas were confirmed by histopathology (data not shown). The total DNA was extracted from fragments of tumors or normal bladder tissues weighing 25 mg by using a Qiagen DNeasy Blood and Tissue kit (Qiagen, Hilden, Germany).

### Primer Design for SYBR Green-Based Quantitative PCR

To detect the BPV2 and 13 DNA, the nucleotide sequences of the moderately variable E6 gene of these viral types were selected to design two sets of specific primers using Primer Blast software. These primer pairs were able to amplify approximately 120 bp of the E6 ORF (Table 1). Two positive controls (17), one of each BPV type, were amplified using the designed primers. Two conventional PCRs were performed to test these specific E6 primers, and the cycling conditions consisted of an initial denaturing step at 94°C for 5 min followed by 35 cycles at 94°C for 1 min, 60°C for 30 s and 72°C for 1 min, followed by a final extension at 72°C for 7 min.

**Table 1:** Primer sets designed to amplify the E6 ORF in BPVs 2 and 13.

**Primer**	**Sequence (5**^**′**^**-3**^**′**^**)**	**Position**^**a**^
BPV2E6fw	GTTTGGTGCAGGGAGCC	143–158
BPV2E6rv	TCTAAGCAGTTCTCAAGACAAG	246–266
BPV13E6fw	CTCTGGTGCAAAGAGCC	144–159
BPV13E6rv	CCTTCAAGACAAGGGGTG	246–258

a *Based on BPV2 and BPV13 reference sequences (GenBank accession number M20219 and JQ798171, respectively)*.

### Production of Standard Plasmid

The E6 gene fragments of each BPV type were cloned using a pCR^™^ 4-TOPO^®^ vector and transformed into *Escherichia coli* TOP10 according to the manufacturer's instructions (Invitrogen Life Technologies, Carlsbad, CA, USA). The recombinant plasmids were extracted using a PureLink Quick Plasmid Miniprep Kit (Invitrogen Life Technologies). To confirm the presence of the E6 gene fragment in the plasmids, direct nucleotide sequencing was performed by using a BigDye Terminator v.3.1 Cycle Sequencing Kit (Applied Biosystems, Foster City, CA, USA) with the corresponding forward and reverse primers in a 3500 Genetic Analyzer (Applied Biosystems). The recombinant plasmids with E6 fragments were quantified using a Quant-iT dsDNA BR Assay Kit in a Qubit Fluorometer (Invitrogen Life Technologies).

### Quantification PCR Using SYBR Green

To quantify the E6 genes in BPVs 2 and 13, two qPCR assays were performed using 10 μL of Platinum SYBR Green qPCR SuperMix (Invitrogen Life Technologies), 20 μM of each primer, and 0.1 μL of ROX, in a final volume of 20 μL in 7500 Fast Real-Time PCR (Applied Biosystems). The following thermal profile was used: polymerase activation at 95°C for 10 min followed by 40 amplification cycles of 10 s at 95°C and 1 min at 60°C each (annealing-extension step).

### Limit of Detection and Specificity of the Assays

To determine the limit of detection (LOD) of the assay, six 10-fold serial dilutions of the BPV2 plasmid containing 1.14 × 10^6^ down to 1.14 × 10^1^ DNA copies/μL and the BPV13 plasmid containing 1.54 × 10^6^ down to 1.54 × 10^1^ DNA copies/μL were analyzed by conventional PCR. The same dilutions were tested in duplicate three different times to evaluate the coefficients of variation (CVs) of the qPCR. The intra- and interassay CVs for the quantification cycle (Cq) values were calculated. The LOD of the qPCR performed using SYBR Green was compared to the conventional PCR assay using the same primer sets. To test the specificity of both PCR assays, the BPV2 and 13 standard curves were run under optimal assay conditions using appropriate primers (specific BPV2 primers in BPV13 samples and specific BPV13 primers in BPV2 samples). A negative control was also included in all the assays.

### Conventional PCR to Screening Test and Sequencing Analyses

To screen the tissue samples, a conventional PCR was performed that targeted a common sequence of the E5 L2 open reading frames (ORFs) (18). PCR products resulting from the conventional amplification assay were purified using a PureLink Quick Gel Extraction and PCR Purification Combo Kit (Invitrogen Life Technologies). Direct sequencing was then performed using a BigDye Terminator v.3.1 Cycle Sequencing Kit (Applied Biosystems) with the respective primers in a 3500 Genetic Analyzer (Applied Biosystems) according to the manufacturer's instructions. The resulting sequences were examined with PHRED software for a quality analysis of the chromatogram readings. Consensus sequences were determined using CAP3 software, and the sequence identity was verified against all the sequences deposited in GenBank using BLAST software.

## Results

### SYBR Green qPCR Standard Curve and Dynamic Range

**Figure 1:**
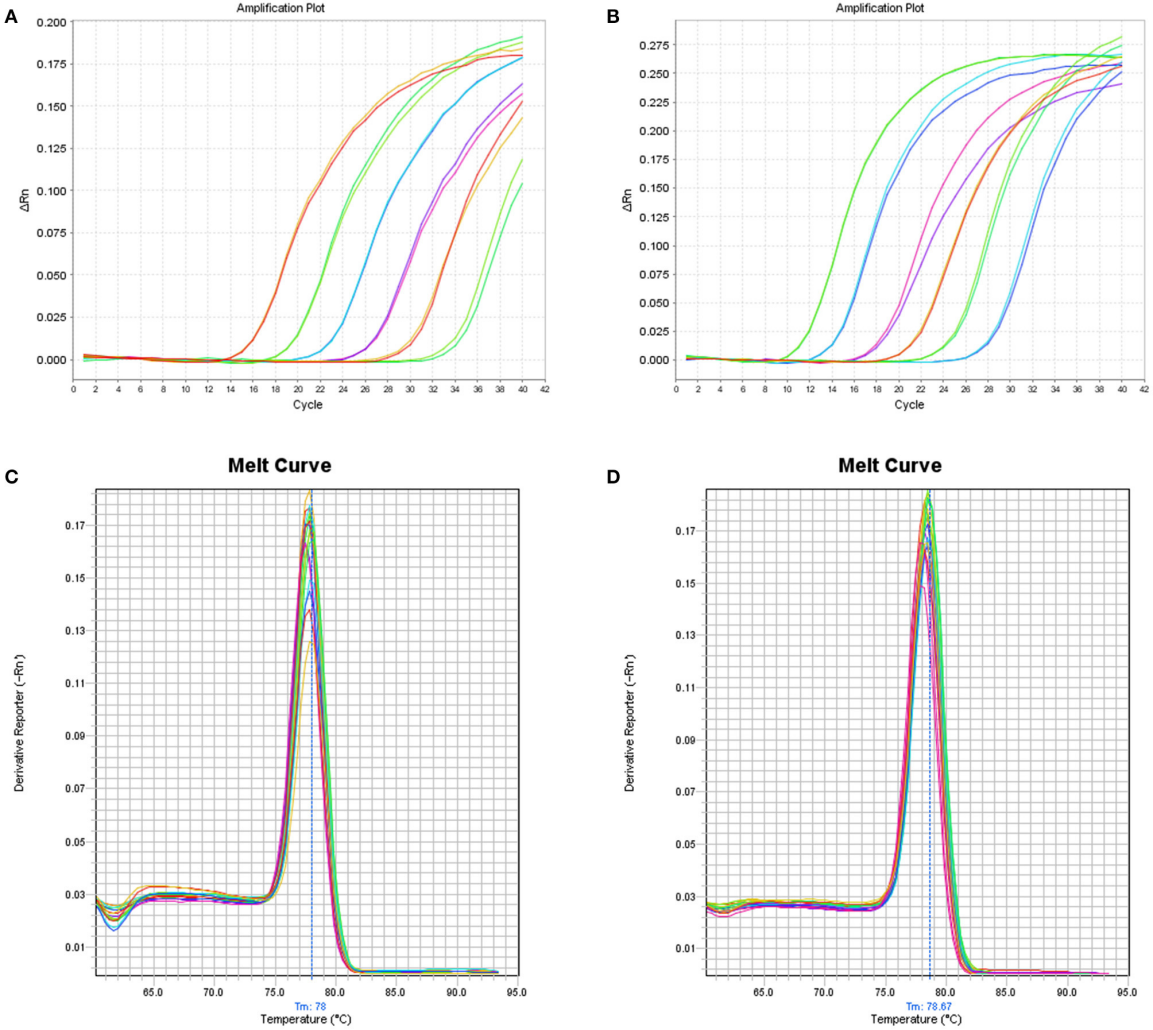
**(A)** Linear amplification curves of the SYBR Green-based qPCR assay using six duplicated 10-fold serial dilutions (10^6^-10^1^ copies/μL) of the BPV2 plasmid template. The standard curves for BPV2 present *R*^2^ = 0.999, slope = −3.488, and eff = 98.2%. **(B)** The linear amplification curves of the SYBR Green-based qPCR assay using six duplicated 10-fold serial dilutions (10^6^-10^1^ copies/μL) for the BPV13 plasmid. The standard curves for BPV13 present *R*^2^ = 0.998, slope = −3.698, and eff = 96.7%. **(C,D)** Melt curves of the BPV2 and BPV13 qPCR assays, respectively.

Six 10-fold serial dilutions of BPV2 and 13 plasmids were used to generate a standard curve by plotting the logarithm of the plasmid copy number against the measured Cq values (Figure 1). The BPV2 Cq values ranged from 15.99 to 32.56 cycles with a linear correlation (*R*^2^) of 0.999 (slope = −3.488) between the Cq value and the logarithm of the BPV2 copy number (Table 2). The BPV13 Cq values ranged from 13.03 to 30.51 cycles with a linear correlation (R^2^) of 0.998 (slope = −3.698) between the Cq value and the logarithm of the BPV13 copy number (Table 2). The efficiency of the two qPCR assays was 98.2 and 96.7%, respectively.

**Table 2:** Intra- and interassay variability of SYBR Green qPCR for BPV2 and 13 E6 gene detection.

**BPV**	**Viral copies**	**Cq mean**	**Intra assay**^**a**^		**Inter assay**	
			**SD**	**%CV**	**SD**	**%CV**
Type 2	1.14 × 10^6^	15.99	0.05	0.27	0.61	3.81
	1.14 × 10^5^	19.46	0.03	0.15	0.87	4.46
	1.14 × 10^4^	22.02	0.02	0.06	2.20	9.99
	1.14 × 10^3^	25.45	0.09	0.33	2.41	9.48
	1.14 × 10^2^	28.97	0.16	0.52	2.54	8.77
	1.14 × 10^1^	32.56	0.37	1.07	2.38	7.32
Type 13	1.54 × 10^6^	13.03	0.02	0.15	0.84	6.44
	1.54 × 10^5^	16.33	0.11	0.63	1.24	7.60
	1.54 × 10^4^	19.96	0.01	0.03	0.81	4.05
	1.54 × 10^3^	23.45	0.01	0.03	1.31	5.61
	1.54 × 10^2^	26.86	0.11	0.39	1.39	5.20
	1.54 × 10^1^	30.51	0.88	2.78	1.45	4.76

a *Relative to the third test value*.

*SD, Standard deviation*.

*%CV, Percentage of coefficient variation*.

### Detection of Conventional PCR Parallel to qPCR

The LOD of the conventional PCR and qPCR employing primer pairs designed to detect the E6 oncogene were evaluated by testing six 10-fold serial dilutions of the DNA standards, which contained 1.14 × 10^6^ down to 1.14 × 10^1^ DNA copies/μL of BPV2 and 1.54 × 10^6^ down to 1.54 × 10^1^ DNA copies/μL of BPV13. A quantitative analysis of the conventional PCR identified LODs of ~114 and 154 copies of BPV2 and 13 DNA, respectively. The qPCR identified 11.4 and 15.4 copies of BPV2 and 13 DNA, respectively.

### Specificity and Reproducibility of qPCR

The positive controls for both viruses were detected using the qPCR assays and the conventional PCRs employing the same primer pairs targeting the BPV E6 oncogene. No cross-reaction was observed when primers specific for the BPV2 E6 gene were tested against BPV13 DNA or vice versa. The reproducibility was analyzed using six 10-fold serial dilutions of a standard for BPVs 2 and 13. The BPV2 qPCR intra-assay CVs ranged from 0.065 to 1.077%, while the interassay CVs ranged from 3.81 to 9.99% (Table 2). The BPV13 qPCR intra-assay CVs ranged from 0.028 to 2.77%, and the interassay CVs ranged from 4.04 to 7.6% (Table 2). A nucleotide sequence analysis of the ~120 bp amplicons confirmed the BPV types in the amplified products.

### Analyses of Clinical Samples by Conventional and New qPCR Strategies

Through PCR using the E5L2 primers, amplicons of the expected length, measuring ~250 bp, were amplified from 8 out of 12 bladder tumor samples. The direct sequencing of the resulting E5L2 products demonstrated the presence of BPV2 in six bladder tumor samples and the identification of BPV13 DNA in two samples of bladder tumors. The nucleotide sequence identity for the E5L2 fragments varied from 98.6 to 100% for BPV2 and 99.8 to 100% for BPV13.

In the analyses of the clinical samples by SYBR Green qPCR as developed in this study, six out of 12 tumor samples presented BPV2 DNA at concentrations ranging from 1.05 × 10^4^ to 9.53 × 10^3^ copies/μL, and two bladder samples presented BPV13 DNA at a concentration of 1.30 × 10^4^ to 1.23 × 10^4^ copies/μL. The 12 healthy bladder mucosa samples were negative for both BPVs. Nucleotide sequence analyses of the ~120 bp amplicons confirmed the BPV types in the amplified products.

## Discussion

In this study, a new real-time SYBR Green-based qPCR system was developed and tested for the detection and quantification of the E6 ORF of BPV types 2 and 13.

The qPCR strategy developed herein presented high sensitivity, specificity, reproducibility, and efficiency for the qualitative and quantitative detection of the E6 oncogene of BPVs 2 and 13 in clinical specimens from affected animals, despite the close phylogenetic relationship among these BPV types (19). Once this approach represents the first qPCR technique for the detection and quantification of BPV13 DNA, the use of this molecular tool will aid in testing diverse clinical samples from a high number of animals suspected of suffering from infections with these viral types, both in single and double infections, at a lower cost and without the need for direct sequencing to confirm the BPV type identities.

BPVs 2 and 13, which are classified as members of the *Deltapapillomavirus* genus of the *Papillomaviridae* family, are involved in the benign growth of the cutaneous epithelium as well as in malignant lesions present in the mucosa of cattle. Among Delta-PVs, BPV2 is the viral type most frequently associated with the carcinogenesis of the urinary bladder of cattle who feed on bracken ferns (2, 6, 12, 16). However, BPV13 was also implicated as a possible causative factor of urothelial tumors in cattle both in single infections and in coinfecting urinary bladder tissue with BPV2 in an investigation performed more recently in the southern region of Italy, where a high rate of BPV13 DNA and E5 oncoprotein expression was demonstrated in tumoral tissues (8). Despite the common occurrence of chronic BEH in cattle from several geographic regions around the world that are known for being endemic with this disease, the identification of an association of BPV13 DNA with clinical specimens collected from the affected cattle is still scarce.

In Brazil, where cattle breeding is hampered in several regions due to the endemicity of BEH, previous investigations on the participation of BPV in the etiology of urinary bladder cancer confirmed the presence of BPV2 DNA through conventional specific PCR assays (6, 20). In the present study, the detection of the BPV2 E6 oncogene in urinary bladder lesions agrees with findings from previous studies. Besides, for the first time in Brazil, the identification of BPV13 DNA in urinary bladder tumors from cattle suffering from this clinical syndrome is reported and agrees with data obtained during a previous investigation on cattle grazing on lands infested with bracken ferns from southern Italy (8). Additionally, by testing DNA purified from normal urinary bladder tissue of healthy cattle from the same endemic area in southern Brazil through our SYBR green-based qPCR, we did not detect the presence of BPVs 2 and 13. Another study analyzing urinary bladders without macroscopic lesions through BPV2 L1 gene-specific conventional PCR previously demonstrated a low rate (10%) of detection of BPV2 DNA in asymptomatic animals from a bracken fern-free region (6).

In this study, four out of 12 malignant lesions of the urinary bladder, histologically confirmed as carcinomas, tested negative for BPV2 and BPV13 DNA in the novel qPCR assay. In a recent molecular investigation of 39 urothelial tumors from cattle suffering from BEH from southern Italy, for the presence of BPV2 and BPV13 DNA through conventional PCR techniques, five carcinomas also tested negative for both viral types (8). Once urinary bladder tumors in cattle not infected by BPV or not exposed to pastures infested by bracken fern are considered extremely rare, accounting for only 0.01–0.1 percent of all bovine cancers, a plausible explanation for the absence of detection of BPV2 and BPV13 DNA in these lesions could be the presence of other Delta-BPVs in such tumors, unable to be recognized by the primer pairs herein designed and employed (21). Recently, BPV14 DNA, another viral type recognized to belong to the *Deltapapillomavirus* genus, was detected in urothelial tumors from cattle from Italy, mostly accompanied by BPV2 and/or BPV13 DNA in multiple infections, being the BPV14 DNA identified in a single infection in only a tumor sample (22). However, studies aiming to confirm the BPV14 involvement in bladder tumor development, demonstrating the pathogenic potential of this viral type by studying the presence of BPV14 mRNAs and expression of viral proteins in urothelial tumors of cattle were not performed. Since in the last years a considerable increment in the number of new BPV types that were characterized was observed, another alternative explanation for these findings could be the occurrence of other unreported Delta-BPVs in urinary bladder tumors, unable to be detected by the qPCR system developed (23).

To the authors, the main limitation of this study was the restricted number of bovine urothelial tumors examined by the novel qPCR system which hampered the elucidation of the frequency of naturally occurring malignant bladder tumors presenting single or multiple infections by BPVs types 2 and 13 in cattle presenting BEH from southern Brazil. However, further investigations aiming to evaluate a larger number of samples from urothelial tumors and healthy urinary bladders for the presence of BPV 2 and BPV13 DNA are needed to be performed in cattle from endemic areas for BEH in Brazil.

In conclusion, the new real-time quantitative PCR assay developed to detect and quantify the E6 oncogene in BPVs 2 and 13 may be a useful molecular tool for diagnostic purposes in clinical cattle samples, allowing for epidemiological investigations on the prevalence of these BPV types in diverse geographical areas as well as their association with different clinical outcomes. This assay thus contributes to studies on the pathogenesis of BPV and the consequent development of preventive and curative strategies to control important diseases due to BPV infections that cause severe economic losses in the cattle industry.

## Data Availability Statement

The raw data supporting the conclusions of this article will be made available by the authors, without undue reservation.

## Ethics Statement

Ethical review and approval was not required for the animal study because the biological samples employed in the study were collected from slaughtered cattle from a private abattoir.

## Author Contributions

BKA carried out most of the experiments, with the collaboration of AMDA. BKA and ML analyzed the data and wrote the manuscript. AAA and AFA procured funding and supervised the project. All authors contributed to the article and approved the submitted version.

## Conflict of Interest

The authors declare that the research was conducted in the absence of any commercial or financial relationships that could be construed as a potential conflict of interest.
